# Transcriptional and Metabolic Profiling of *Arabidopsis thaliana* Transgenic Plants Expressing Histone Acetyltransferase *HAC1* upon the Application of Abiotic Stress—Salt and Low Temperature

**DOI:** 10.3390/metabo13090994

**Published:** 2023-09-05

**Authors:** Tatiana Ivanova, Ivayla Dincheva, Ilian Badjakov, Anelia Iantcheva

**Affiliations:** AgroBioInstitute, Agricultural Academy, Blvd. Dragan Tzankov 8, 1164 Sofia, Bulgaria; titi_986@abv.bg (T.I.); ivadincheva@abi.bg (I.D.); ibadjakov@gmail.com (I.B.)

**Keywords:** *HAC1* histone acetyltransferase, *A. thaliana* transgenic plants, abiotic stress salinity and low temperature, transcript analyses, metabolic analysis, free metabolites, amino acids, sugars, fatty acids

## Abstract

Augmented knowledge of plant responses upon application of stress could help improve our understanding of plant tolerance under abiotic stress conditions. Histone acetylation plays an important role in gene expression regulation during plant growth and development and in the response of plants to abiotic stress. The current study examines the level of transcripts and free metabolite content in transgenic *Arabidopsis thaliana* plants expressing a gene encoding histone acetyltransferase from *Medicago truncatula* (*MtHAC1*) after its heterologous expression. Stable transgenic plants with *HAC1* gain and loss of function were constructed, and their T_5_ generation was used. Transgenic lines with *HAC1*-modified expression showed a deviation in root growth dynamics and leaf area compared to the wild-type control. Transcriptional profiles were evaluated after the application of salinity stress caused by 150 mM NaCl at four different time points (0, 24, 48, and 72 h) in treated and non-treated transgenic and control plants. The content and quantity of free metabolites—amino acids, mono- and dicarbohydrates, organic acids, and fatty acids—were assessed at time points 0 h and 72 h in treated and non-treated transgenic and control plants. The obtained transcript profiles of *HAC1* in transgenic plants with modified expression and control were assessed after application of cold stress (low temperature, 4 °C).

## 1. Introduction

Transcriptional regulation in eukaryotes is not simply determined by DNA sequence but is mediated by chromatin modifications and remodeling. Dynamic changes in histone protein acetylation induce specific variations in gene expression. It influences different biological processes in response to internal and external signals, including cell differentiation, growth, development, light, temperature, and abiotic and biotic stress [1].

In eukaryotic cells, DNA is packaged with histones and forms a complex structure known as chromatin. The structural unit of chromatin is the nucleosome, which consists of approximately 147 base pairs of DNA wrapped around a histone octamer containing two molecules of each histone protein—H2A, H2B, H3, and H4—linked together by protein–protein interactions and a single linker histone H1. Histone H1 is a binding structure that stabilizes the octamer. The amino termini of histones possess positively charged lysine (lys+) and arginine (arg+) amino acids, which cause affinity between positively charged histones and negatively charged DNA [2,3]. The N-terminal tails of histones “protrude” from the nucleosome and are subjected to post-translational modifications, such as acetylation and deacetylation [4,5]. The N-terminal lysine residues of histone H3 (K9, K14, K18, K23, and K27) and H4 (K5, K8, K12, K16, and K20) are the main targets for acetylation/deacetylation in plants [6]. Acetylation and deacetylation of histones are catalyzed by histone acetyltransferases (*HATs*) and histone deacetylases (*HDACs*). Hyperacetylation of histones is associated with transcriptional activation, whereas hypoacetylation of histones induces chromatin compaction and gene repression [7,8]. Accumulated literature data show that plant *HATs* and *HDACs* play essential roles in the regulation of gene expression, plant development, and plant responses to environmental stress [9].

*HATs* are classified into two main classes: *HAT*-A and *HAT*-B [10,11]. *HAT*-A is localized in the cell nucleus, and its function is to acetylate the core histones of the nucleosome. It acts as a transcriptional coactivator and is therefore important for gene expression.

The *HAT*-A class is divided into five families: GCN5-linked N-terminal acetyltransferases (GNATs), MYST (MOZ, Ybf2/Sas3, Sas2, and Tip60)-linked HATs, p300/CREB binding protein (CBP), and initiation factor transcription TAFII 250 [for TATA-binding protein (TBP)-binding factor]. The *Arabidopsis* genome contains five p300/CBP (p300/CREB-binding protein) genes: *AtHAC1*, *AtHAC2*, *AtHAC4*, *AtHAC5,* and *AtHAC12,* and two TAFII250 genes: *HAF1* and *HAF2* [2]. 

Plants are fixed and immobile organisms that cannot choose their environment. It is essential for plants to develop rapid responses to changing environmental conditions in order to adapt and survive. The control of chromatin modification may play a central role in the regulation of gene expression in response to environmental cues. The switch from acetylated to deacetylated chromatin and vice versa enables the alteration of gene expression. A number of studies have shown that plants are able to adapt their growth and development to environmental changes such as light, temperature, and biotic and abiotic stress by modulating histone acetylation [12,13]. Over the past two decades, a lot of research has been undertaken to decode plant responses to biotic and abiotic stress. More recent evidence indicates that chromatin modification plays an essential role in the epigenetic regulation of gene expression and the adaptation of plants to stress [14,15,16].

Some histone modifications occur rapidly in response to environmental changes. Others occur gradually along with changes in gene expression to control physiological homeostasis and development under environmental stress [17,18]. Alteration in expression by acetylation of histones in response to stress has been investigated in different plant species. For example, salinity, drought, and heat stress increase the acetylation levels of histone H3 lysine 9 (H3K9ac) and histone H4 lysine 5 (H4K5ac) in various maize tissues [19,20,21,22]. In rice seedlings, drought stress induces acetylation of H3K9, H3K18, H3K27, and H4K5ac [23]. In *Arabidopsis*, the N-terminal lysine residues K9, K14, K18, K23, and K27 of histone H3 and K5, K8, K12, K16, and K20 of histone H4 were found to be targets for acetylation/deacetylation [6]. The increased level of histone H3 acetylation was observed after exposure to salinity or wounding, whereas drought stress did not affect histone H3 acetylation. Region-specific acetylation is associated with a region-specific change in the chromatin structure to increase the accessibility of stress-responsive transcription factor binding sites. Differential acetylation of H3K18 and H3K27 lysine residues may be involved in transcriptional regulation under cold stress [24]. Higher acetylation activities or lower deacetylation activities are induced during stress responses. HAT enzymes possess preferential targets of lysine in histone H3 or H4 [25,26,27], but acetylation could extend to adjacent lysine residues [28]. When the primary metabolism is strongly influenced by stress or growth conditions, metabolic control of histone acetylation is possible. Increasing histone acetylation is generally believed to play an essential role in the de novo programming of gene expression for plant responses to stress. However, the increase in histone acetylation at some loci may lag behind gene activation. In this case, it is a consequence rather than a cause of stress-induced gene expression [29]. 

In this study, we analyzed the *HAC1* gene, which belongs to the *HAT*-A class acetyltransferases p300/CREB family and is located in the cell nucleus and acetylates the core histones [30]. Little is known about the role of *HAC1* in the development of *A. thaliana* and other plant species, while its animal homolog, p300/CREB, is well characterized [31]. The *HAC1* investigated by us was originally identified by a reverse genetic approach in a population of *Tnt1* retrotransposon-tagged mutants of the model legume *Medicago truncatula*. After sequencing some mutant lines, an insertion of a *Tnt1* transposon in an exon in the coding region of the histone acetyltransferase gene (*HAC1*) was found [32]. Seeds from *Arabidopsis thaliana* wild-type (WT, control) and T_5_ homozygous transgenic lines with overexpression of the *HAC1* gene (*HAC1*^OE^) or downregulation (*HAC1*^RNAi^) were used. The transgenic lines were constructed and described in our previous research [30]. In the current investigation, the transcriptional and metabolite profiles of *A. thaliana* lines with *HAC1* gain (*HAC1*^OE^) and loss (*HAC1*^RNAi^) of function and control plants were collected and evaluated under conditions of abiotic stress such as salinity and low temperature. The accumulated results from experiments were analyzed and discussed.

## 2. Material and Methods

### 2.1. Plant Material and Maintenance

Wild-type control *A. thaliana* ecotype Columbia-0 (Col-0) seeds and T_5_ seeds from the *A. thaliana* transgenic lines (*HAC1^OE^* and *HAC1^RNAi^*) were used for the experimental work. The *A. thaliana* transgenic lines were constructed and described in detail in our previous research [30]. Briefly, the procedure for generating *HAC1* constructs was based on the Gateway cloning system (Invitrogen Life Technologies, Inc., Warrington, UK, www.lifetechnologies.com accessed on 1 September 2009). For the overexpression plants, *HAC1* from *M. truncatula* was heterologously expressed in *A. thaliana*. To design the entry clones, the open reading frame (ORF) of the *M. truncatula HAC1* gene (*MT0G02950*, Plaza 2.5, www.medicago.org accessed on 1 September 2009) was cloned into the *pDONR221* donor vector. The *HAC1* gene entry clone was transferred into the destination vector for overexpression (*pK7WG2*) under the control of the CaMV 35S promoter and the *nptII* gene for plant selection [33]. 

To generate constructs for downregulation, the RNA interference (RNAi) strategy [34] was applied. To create an expression clone, the binary Gateway vector *pK7GWIWG2D(II)* for hairpin RNA expression was used. A fragment of 128 bp, corresponding to nucleotide positions 138–264 of the ORF of the *Arabidopsis* ortholog *At1g79000* was targeted. The generated constructs were introduced into *Agrobacterium tumefaciens* strain C58C1. Stable *A. thaliana* transgenic lines with *HAC1* overexpression (OE) and RNAi-mediated knockdown (RNAi) were constructed by *A. tumefaciens*-mediated transformation via the floral-dip method [35]. The T_5_ homozygous generation of the transgenic lines was used for the experimental work.

The seeds from control and transgenic lines were sterilized by using 96% ethanol for 1 min, followed by bleach solution for 12 min, and then washed at least 4–5 times with sterile distilled water. Germination was carried out on basic MS_0_ medium [36] in square Petri dishes. Petri dishes were placed for 48 h in the dark at 4 °C to synchronize germination and subsequently transferred to a cultivation room. The obtained in vitro plants were cultivated in a climate room under standard conditions: temperature around 23–24 °C, 70–80% relative humidity, photoperiod 16/8 h (day/night), intensity of lighting 150–300 m^−2^ s^−1^. In order to obtain seeds and evaluate the plant morphology, 15-day-old in vitro seedlings were transferred to the adaptation room and planted in soil in cultivation trays (24 holes). A protector was placed on each individual plant.

### 2.2. Morphometric Analyses of A. thaliana HAC1^OE^ and HAC1^RNAi^ Transgenic Lines and Control Plants

The morphometric analyses were carried out on transgenic and control plants not subjected to stress by tracking the following parameters: root growth dynamics and leaf area. 

#### 2.2.1. Dynamics of Root Growth

Seeds of transgenic plants with modified *HAC1*^OE^ and *HAC1*^RNAi^ expression and control were sterilized and placed on basal medium (MS_0_) in square Petri dishes. After a period of 48 h at 4 °C in order to synchronize seed germination, the Petri dishes were transferred to a climate room under controlled conditions and grown in a vertical position. The primary root length was taken after 5 days, and the increase in the length of the main root was recorded after each 24th, 48th, 72nd, 96th, and 120th h. Measurements of at least 40 roots of the pant line were made using the software program Image J v1.4.3 (Rasband WS, USA National Institute of Health, Bethesda, MD, USA). (Supplementary Figure S1)

#### 2.2.2. Measurement of Leaf Area

Leaf area was determined as the mean sum of 6 individual areas of each line (OE, RNAi, and WT) detached from the rosette of plants grown under greenhouse conditions (Supplementary Figures S2 and S3). The collected plant material was placed in square Petri dishes and scanned. Measurements of 2 sets of 6 individual areas were made using the software program Image J v1.4.3 (Rasband WS, USA National Institute of Health, Bethesda, MD, USA).

### 2.3. Treatment of Seedlings of A. thaliana HAC1^OE^ and HAC1^RNAi^ Transgenic Lines and Control with 150 mM NaCl

A square piece of filter paper soaked in a 150 mM NaCl solution was placed in a square Petri dish with basal medium (MS_0_). Twenty 15-day-old *A. thaliana* seedlings, with modified expression (*HAC1*^OE^ and *HAC1*^RNAi^) and control, were placed on the soaked filter paper. The experiment was conducted twice for the time intervals (0, 24, 48, and 72 h). At each time point, samples were taken to assess the expression level of the *HAC1* gene using quantitative qRT-PCR analysis.

### 2.4. Cold Treatment of A. thaliana HAC1^OE^ and HAC1^RNAi^ Transgenic Lines and Control

To carry out the experiment, mature *A. thaliana* plants *HAC1*^OE^ and *HAC1*^RNAi^ and WT (2 sets of three plants per line) were placed in a refrigerator at a temperature of 4 °C. Plants were subjected to the stress factor for a period of time—0 and 72 h—due to their inability to survive longer under conditions of low temperature. For each time interval, leaf samples from the rosettes were taken, and the expression level of the *HAC1* gene was assessed (Supplementary Figure S4).

### 2.5. Expression Analyses

#### 2.5.1. Isolation of Total RNA

Total RNA was isolated from 15-day-old seedlings treated and non-treated with NaCl. Leaves from the rosettes of plants with modified expression (*HAC1*^OE^ *HAC1*^RNAi^) and control (WT) treated and non-treated with low temperatures were also collected. All of the samples were frozen in liquid nitrogen, then homogenized in mortars and stored at −80 °C. RNA was isolated with a kit (GeneMATRIX Universal RNA/miRNA Purification Kit, EURX) following the manufacturer’s protocol. The concentration and quality of isolated RNA were checked on a NanoDrop spectrophotometer. The resulting RNA samples were stored at −80 °C.

#### 2.5.2. Synthesis of cDNA

One microgram of RNA was used for cDNA synthesis. A Bio-Rad kit (iScript cDNA Synthesis Kit, Berkeley, CA, USA) was used. The final volume of the reaction was 20 µL, and the synthesis was performed on a thermal block (LKB-CH 100). The cDNA obtained was stored at −20 °C.

#### 2.5.3. Quantitative qRT-PCR Analysis

By quantitative real-time PCR analysis (qRT-PCR), the expression of the studied gene (*HAC1*) was determined in plants under stress and compared with the expression in control plants. Each reaction has a final volume of 20 µL, including 1:7 cDNA:MQ H_2_O, gene-specific primers, and SYBR Green Mix (EURX). Each sample was tested with three technical repeats (two biological repeats) by dropping the prepared mixes into a 96-well microplate (Applied Biosystems, Waltham, MA, USA) sealed with optical adhesive film (Applied Biosystems). The analyses were carried out on a 7300 Real-Time PCR Systems apparatus from Applied Biosystems. Actin (*ACTIN2*) and ubiquitin (*UBIQUITIN10*) were used as reference genes in order to normalize the Ct value. The obtained values of the studied gene were tracked and averaged. The obtained data were processed with a specialized program, *qBASE v 1.3.5.* (Center for Medical Genetics, Ghent University Hospital, http://medgen.ugent.be/qbase accessed on 10 February 2012). The sequence of the primers used is presented in Supplementary Table S1. The length of the amplicon of primers for *HAC1^OE^* samples is 180 bp, and for *HAC1^RNAi^* samples it is 152 bp. The products obtained from amplification with primers are presented on gels (Supplementary Figure S5).

### 2.6. Metabolic Analysis

Polar and non-polar metabolites were determined by gas chromatography–mass spectrometry (GC–MS) in *A. thaliana* seedling samples from *HAC*^OE^ and *HAC*^RNAi^ lines with modified expression and control treated and non-treated with 150 mM NaCl at time points 0 h and 72 h. Samples in three replicates were analyzed as described earlier [37,38]. The identification of the metabolites was obtained by comparing the retention times and RI with those of authentic compounds and the spectral data obtained from the Golm Metabolome Database (GMD) [39] and the National Institute of Standards and Technology (NIST 08) libraries [40]. The quantitative data (µg/g dried weight) are presented in Table S2.

### 2.7. Statistical Analyses

Experiments were performed with two or three biological repeats. The data from the experiment were evaluated by analysis of variance using Excel software. Results were considered statistically significant at *p* values ≤ 0.05, and data are presented as the mean ± (SD) standard deviation or ±(SE) standard error.

## 3. Results

### 3.1. Root Growth Dynamics in HAC1^OE^ and HAC1^RNAi^ Lines and Control of A. thaliana Not Subjected to Abiotic Stress

**Figure 1:**
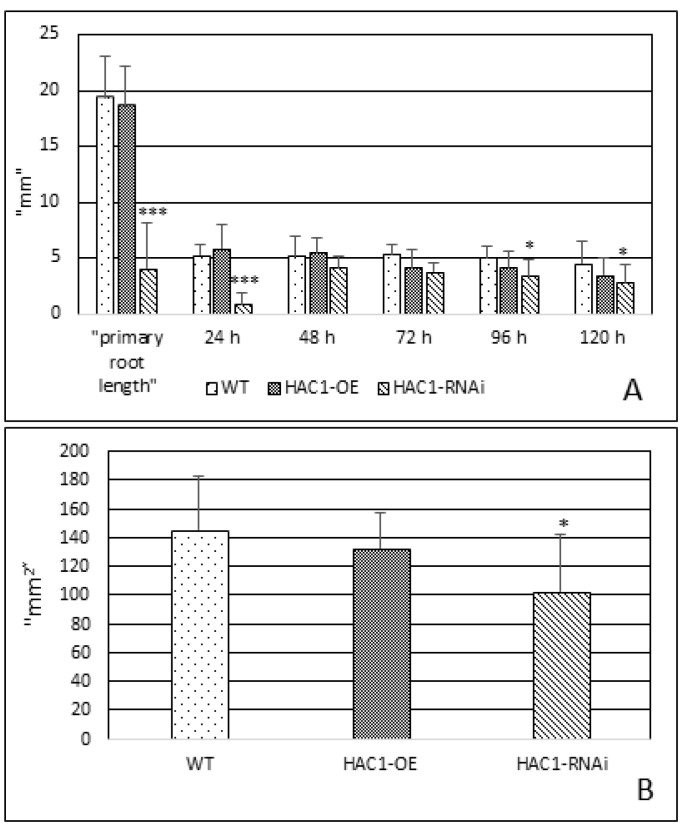
Morphometric analyses of *A. thaliana* transgenic lines with modified expression of the *HAC1* gene (*HAC1*^OE^ and *HAC1*^RNAi^) and control. (**A**) Root growth dynamics; (**B**) size of leaf area. Data represent means ± SD. Asterisks denote statistically significant differences: * *p* < 0.05; *** *p* < 0.001.

The dynamics of root growth were recorded at the primary root of 5-day-old seedlings. Later on, the increase in root length was measured at time points 24, 48, 72, 96, and 120 h (Figure 1A). Statistical analyses on root growth dynamics showed a higher growth value in the *HAC1*^OE^ line at the 24th hour and the 48th hour, compared to the control and *HAC1*^RNAi^ line. In contrast to these results, the *HAC1*^RNAi^ line showed a significant difference in the primary length of the main root and at the 24th hour of growth (*p* ≤ 0.001) compared to those reported in the control and the *HAC1*^OE^ line. A weaker growth rate was observed at the 48th, 72nd, 96th, and 120th hours (*p* ≤ 0.05) compared to the control.

### 3.2. Leaf Area in HAC1^OE^ and HAC1^RNAi^ Lines and Control of Arabidopsis thaliana Not Subjected to Abiotic Stress

Measurements of leaf area in the studied transgenic lines and the control (Figure 1B) showed that both *HAC1*^OE^ and *HAC1*^RNAi^ lines possessed a smaller leaf area compared to the control, and this difference was significant in the leaf area of the *HAC1*^RNAi^ line compared to the control (*p* ≤ 0.05). The results of the measurements are presented in Figure 1B and were determined as the mean sum of individual leaf areas.

### 3.3. Treatment of A. thaliana Seedlings from HAC1^OE^ and HAC1^RNAi^ Transgenic Lines and Control with 150 mM NaCl

**Figure 2:**
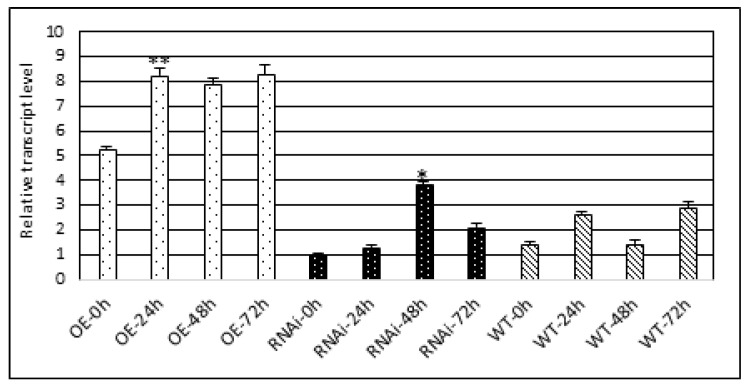
Transcript level of the *HAC1* gene in *A. thaliana* seedlings of transgenic lines with modified expression (*HAC1*^OE^ and *HAC1*^RNAi^) and control treated with 150 mM NaCl solution at four time intervals. Expression data are means ± SE. Asterisks denote statistically significant differences: * *p* < 0.05; ** *p* < 0.01.

The application of abiotic stress salinity was carried out by treatment with a 150 mM NaCl solution. The experiment was performed at four time points—0, 24, 48, and 72 h. Plant samples were taken from each treatment period to evaluate the expression of the *HAC1* gene by performing qRT-PCR (Figure 2). The resulting transcriptional profile showed an increase in gene expression at the 24th hour time point, which was significant (*p* ≤ 0.01) in the *HAC1*^OE^ line and less pronounced in the control, while a slight increase in the transcript level was observed in the line with downregulated expression. At 48 h of NaCl treatment, the *HAC1*^RNAi^ line showed a sharp increase (*p* ≤ 0.05) in the transcript level, while in the *HAC1*^OE^ line and the control, the transcript level was slightly decreased, followed by a slight increase over the 72-hour period. During the 72nd hour of treatment, in the *HAC1*^RNAi^ line, there was a decrease in the transcript level. The response to salinity stress was characterized by a very pronounced increase in the transcript level in the *HAC1*^OE^ line and the maintenance of a high level of expression throughout the treatment period. This pattern of expression was also followed by the control, which leads us to the conclusion about the participation of the studied histone acetyltransferase in the response to salt stress. In the *HAC1*^RNAi^ line, a delay in the response to the applied stress was observed. A significant increase in expression was observed at 48 h compared to time points 0 and 24 h.

### 3.4. Determining the Metabolite Profile of Transgenic Plants with Modified Expression and Controls Non-Treated for 0 h and Treated with 150 mM NaCl for 72 h

**Figure 3:**
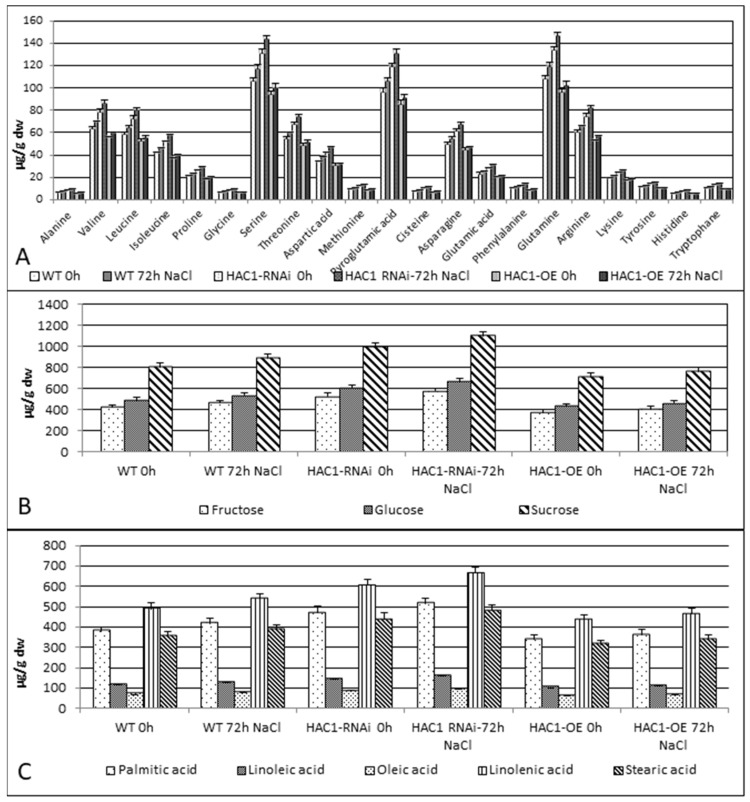
Primary metabolite content of *A. thaliana* transgenic lines with modified expression (*HAC1*^OE^ and *HAC1*^RNAi^) and control non-treated 0 h and after application of 72 h salt stress (150 mM NaCl). (**A**) Detected free AAs; (**B**) some of the detected free sugars; and (**C**) detected free FAs. The quantity of metabolites is presented in µg/g DW, and the data represent means ± SD.

In the course of the experimental work, a metabolic analysis was carried out to determine the amounts of detected free amino acids, organic acids, sugars, and fatty acids. Gas chromatography with mass spectrometry (GC/MS) was used for the purpose of the experiment. The experiment was performed in triplicate, and the results were averaged. The determined amounts of free metabolites were summarized and presented in µg/g DW (Figure 3A–C and Supplementary Table S2). The highest values of the amounts of the free metabolites detected were reported in *HAC1*^RNAi^ plant samples untreated and treated with NaCl, followed by metabolite values reported in controls. The lowest values of the amounts of free metabolites were reported for *HAC1*^OE^ plant samples (Figure 3A–C). 

### 3.5. Treatment with Low Temperature of A. thaliana Plants with Modified Expression HAC1^OE^, HAC1^RNAi^, and Control

**Figure 4:**
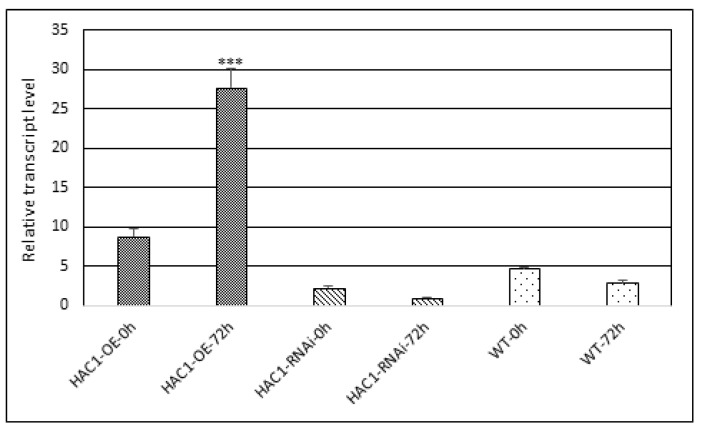
Transcript level of the *HAC1* gene in transgenic plants with modified expression and control from *A. thaliana* upon application of abiotic stress—low temperature. Expression data are means ± SE. Asterisks denote statistically significant differences: *** *p* < 0.001.

In our study, another factor causing abiotic stress was applied—low temperature treatment (cold stress). Tracking the change in the level of expression of the *HAC1* gene in response to cold was carried out with *A. thaliana* transgenic plants with modified expression and a control. Mature *A. thaliana* plants were subjected to a low temperature stress of 4 °C for a period of 72 h. Leaf samples were taken at time points 0 h and 72 h, and the expression level of the *HAC1* gene was assessed (Figure 4). Control and *HAC1*^RNAi^ plants possessed a similar transcriptional profile for the low-temperature treatment period—a decrease in transcript level at the 72nd hour. The gene expression profile of the overexpressing line was characterized by a rapid response to the applied stress and a sharp increase in expression at the 72nd hour (*p* ≤ 0.001). Therefore, the *HAC1*^OE^ plants possessed the highest plasticity to the applied stress, followed by the control. The low-temperature stress experiment on *A. thaliana* was conducted for a period of 72 h due to the plants’ inability to survive a longer stress treatment. After 72 h of low-temperature treatment, plants started to turn yellow and die slowly without flowering or producing seeds. 

## 4. Discussion

The present study is a preliminary investigation of the function of the gene encoding histone acetyltransferase *HAC1* under conditions of induced abiotic stress. Initially, Deng et al., (2007) [31] isolated *Arabidopsis* mutants with T-DNA insertions in the *HAC1* gene and studied their effect on plant development. They found that *HAC1* plays an important role in vegetative and reproductive development. Mutations in At*HAC1* cause multiple defects in plant development, including delayed flowering, a short primary root, and reduced fertility.

Our previously published study on the *HAC1* gene [30] was based on the construction of stable transgenic plants with modified expression of the three model species: two legumes, *Medicago truncatula* and *Lotus japonicus,* and *Arabidopsis thaliana* as a referent plant. We were able to confirm the expression of the *HAC1* gene in different plant tissues and organs characterized by actively dividing cells. Transgenic plants with modified expression of model plants were distinguished by morphological deviations in plant architecture and disturbances in flower morphology, which confirms the role of the gene in plant development. Based on the obtained results, we hypothesize that *HAC1* is involved in the acetylation of the two core histones H2B and H4, typical for the S phase of the plant cell cycle. This was confirmed by the accumulation of their transcripts in plants with downregulated expression of *HAC1*. In the present study, we collected transcriptional and metabolite data from *A. thaliana* plants with gain and loss of function of the *HAC1* gene and controls under the conditions of the applied abiotic stress of salinity and low temperature. The phenotypic evaluation of the greenhouse plants with modified expression and the control not subjected to stress showed almost identical values for the number of leaves in the rosette. The rosettes of the plants with downregulated expression were characterized by leaves of various shapes. The parameters number of branches on the main stems and length of the main stem possess almost the same value in all observed plants. Only for the parameter total number of siliques, a significant difference was observed between the control and plants with downregulated expression (*p* ≤ 0.01), as well as between the number of siliques reported in plants with overexpression and those with downregulated expression (*p* ≤ 0.01). Literature data indicate that mutations in the *HAC1* gene lead to multiple defects such as retarded growth, short primary roots, and reduced fertility [31]. The applied salinity stress led to an increased expression level of the *HAC1* gene significantly in overexpressed plants but not significantly in the control. Even more, the expression was significantly increased after 48 h in plants with downregulation. The results obtained from this experiment are consistent with the results of [19]. The authors reported that in maize, the expression of the *HAT* genes Zm*HATB* and Zm*GCN5* increased after NaCl treatment, accompanied by an increase in the global acetylation levels of histones H3K9 and H4K5. In another study [41], similar results were observed in Chinese cabbage—histone H3K27 and H3K18 acetylation significantly increased upon salt stress treatment for 2 days. During the response to applied salt stress, the transcript levels of Chinese cabbage genes Bra*HAC1*, Bra*HAC2*, Bra*HAC3*, Bra*HAC4*, Bra*HAC5*, Bra*HAC6*, Bra*HAC7*, Bra*HAG1*, Bra*HAG2*, Bra*HAG3*, Bra*HAG5,* and Bra*HAG7* were significantly increased as well. In cotton [42], it was demonstrated that salinity causes a global increase in H3K9 and H3K4 acetylation, and more than one gene was upregulated in response to salt stress. The expression level of Gh*HAC1501*, Gh*HAC1502*, Gh*HAC1503*, Gh*HAG1501*, Gh*HAG1504,* and Gh*HAF1501* was increased compared to the control.

Along with the data collected for the transcript level of the *HAC1* gene during salinity stress, the metabolite analyses were performed after 72 h of stress application. This time point was selected because of the highest expression level of the *HAC1* gene detected in overexpressed lines and the control. The quantity of detected free metabolites—AAs, mono- and dicarbohydrates, and fatty and organic acids—was evaluated in treated and non-treated *HAC1*^OE^ and *HAC1*^RNAi^ lines and controls. The analysis of the obtained results showed a correlation with the function of the *HAC1* gene related to enhanced cell division activity and plant growth. Increased acetylation of histone leads to enhanced gene expression followed by enhanced translation, leading to a reduction in the pool of free AAs and other metabolites, such as sugars and fatty acids (FAs). This assumption correlates with the lowest amounts of the detected free metabolites obtained in *HAC1*^OE^ transgenic lines, followed by the values reported in the control and the highest reported in the *HAC1*^RNAi^ lines. Treatment with 150 mM NaCl resulted in an increase in free metabolites in all samples tested, and a high amount was detected in *HAC1*^RNAi^, followed by control. A smaller increment was detected in the *HAC1*^OE^ samples tested. Results published by [41] showed that histone H3 acetylation was significantly increased upon salt stress treatment for a period of two days and was associated with the expression of stress-responsive genes. After this period, the acetylation of histone H3 and the expression of stress-responsive genes decrease. Most likely, reduced acetylation leads to a decrease in the translation of stress-responsive genes and an increase in the pool of free amino acids and other primary metabolites. 

In this study, changes in primary metabolism after application of salinity stress were detected, including responses in the levels of sugars, amino acids (AAs), and FAs. More pronounced elevated amounts in treated samples of the transgenic and WT plants were detected for the free AAs valine, leucine, isoleucine, serine, threonine, asparagine, glutamine, and arginine. The increased quantity of these free AAs varied from 2.50 µg/g DW to 13.39 µg/g DW for different AAs in all treated samples (Supplementary Table S2). In another study performed with wild and cultivated barley under conditions of salt stress, the authors reported changes in the following amino acids: proline, alanine, aspartate, glutamate, threonine, and valine, where the responses were genotype-specific [43]. In the research with multiple barley varieties exposed to abiotic stress—salt, eight amino acids and amines, 4-hydroxy-proline, asparagine, alanine, arginine, phenylalanine, citrulline, glutamine, and proline—they were detected to be significantly increased [44]. In addition, we detected significant changes in the amount of sugars—sucrose, fructose, and glucose—with more than 50 µg/g DW in treated samples of *HAC1*^RNAi^, WT, and *HAC1*^OE^, compared to non-treated. Fumagalli et al., (2009) [45] studied the metabolite profiles of two different cultivars of rice under salinity (150 mM) and also confirmed enhanced sugar contents during this kind of stress in both of the examined cultivars. The elevated level of sugars detected by us in treated samples is consistent with the role of sugars as osmolytes [46]. The osmolytes play an important role in the preservation of osmotic homeostasis by lowering the osmotic pressure caused by salt stress. In the study of Dias et al., 2015 [47], performed with two chickpea cvs., the response to salt stress was contrasting, and changes were detected in sugar metabolism, the TCA cycle, and amino acid metabolism. One of the metabolites detected in our study with a pronounced amount was pyroglutamic acid (5-oxoproline). This metabolite is an intermediate in the glutamil cycle pathway for the biosynthesis and degradation of glutathione [48]. Glutathione (γ-glutamyl-cysteinyl-glycine) is a small molecule that possesses strong non-enzymatic antioxidant activity. Clearly, glutathione is one of the ways to overcome the applied salt stress in WT and *HAC1*^OE^*/HAC1*^RNAi^ lines. In *Arabidopsis*, heterologous expression of rice glutathione S–transferase (*OsGSTU4*) leads to an increase in tolerance to salt stress [49].

In the collected metabolite data, we also detected saturated and unsaturated FAs. Their amount increased after the application of salinity stress. The increment varied from 3.93 µg/g DW, the lowest, to 60.64 µg/g DW, the highest, for different FAs. In the literature, fatty acids have been shown to be active players in the stress response [50]. They participate in different types of abiotic and biotic stresses and regulate tolerance to salt, drought, and heavy metals, and are involved in responses and defenses against insects [51]. The other possibility for an increment of FAs is the possibility of being a source for ß-oxidation in the peroxisome to produce Acetyl-CoA. Its availability could regulate histone lysine acetylation [29].

In the data collected from our metabolite profile, we did not detect significant levels of organic acids, except gluconic acid. It has been shown that low levels of gluconic acid lactone are related to drought tolerance in broccoli [52]. The elevated level of gluconic acid was detected in the roots of seedlings from wild soybeans under conditions of alkaline slat stress [53]. 

The changes in the surrounding environment caused alterations in the expression of plant genes. Histone acetylation plays an important role in the regulation of gene expression in the plant’s response to abiotic stress. According to different studies, transcriptional activation of stress-related genes is directed in two ways. The first is the synthesis of regulatory proteins (transcription factors, protein kinases, signal enhancers) and functional proteins related to membrane, cell wall, cytoskeleton reorganization, ion transport and channels, and osmolyte and antioxidant biosynthesis. The second way is if stress-expressed genes are related to changes in energy metabolism (nitrogen metabolism, carbohydrate metabolism, cellular respiration, and photosynthesis) [54]. The mechanism of plant tolerance to salt stress is complex and species-specific. In our investigation, we confirmed the role of the *HAC1* gene in the beginning of the plant’s response to induced salt stress. Based on the data on the content of primary metabolites detected in this study, we could direct our further research and select possible gene targets upon application of salt stress.

The significantly elevated level of transcripts in the *HAC1*^OE^ line after 72 h cold treatment confirms the involvement of the studied gene in response to the exact abiotic stress. The role of *HATs* in response to cold is discussed and analyzed in the study performed with rice related to differential acetylation of histone H3 at the regulatory region—a promoter of the stress-responsive gene Os*DREB1b* [24]. Our future intent is to perform metabolic profiling after 72 h of low-temperature stress.

## 5. Conclusions

In response to abiotic stress, plants establish a set of responses in order to avoid the stress and release different mechanisms of defense to cope with it. These defense mechanisms are connected with the reprograming of gene expression, the elevated transcription of defense genes, and enhanced changes in primary metabolism. The results from the established transcriptional and metabolic profiling of *HAC1* transgenic plants with modified expression and controls presented in this study are preliminary and need further large-scale experiments to be thoroughly analyzed. The data collected from this study augmented our knowledge about the function of the *HAC1* gene and its role in the conditions of abiotic stress such as salinity and low temperature.

## Data Availability

All data are available in the manuscript and Supplementary Materials.

## Supplementary Materials

The following supporting information can be downloaded at: https://www.mdpi.com/article/10.3390/metabo13090994/s1, Figure S1: Primary root length of 5 days old seedlings of *A. thaliana* lines with modified expression (HAC1-OE and HAC1-RNAi )and control (WT); Figure S2: Age of rosettes from *A. thaliana* transgenic and control plants for collection of samples from leaf area measurement; Figure S3: Samples for leaf area measurement; Figure S4: *A. thaliana* plants (WT, HAC1-OE and HAC-RNAI) before low temperature (4 °C) stress; Figure S5: Amplification products. (A) amplicon of HAC1-OE sample; (B) amplicon of HAC1-RNAi sample; Table S1: Primers used in qRT-PCR analyses; Table S2: Content and quantity of detected polar and non-polar free metabolites in µg/g DW in non-treated and treated samples.
