# Supplementary material for: Transcriptional and Metabolic Profiling of Arabidopsis thaliana Transgenic Plants Expressing Histone Acetyltransferase HAC1 upon the Application of Abiotic Stress—Salt and Low Temperature
Source: Metabolites. 2023 Sep 5;13(9):994. doi: 10.3390/metabo13090994 (PMC10536276; doi:10.3390/metabo13090994)
Supplement: Supplementary file 1 [file metabolites-13-00994-s001.zip › metabolites-2586379-supplementary.pdf]

Figure S1. Primary root length of 5 days old seedlings of *A.thaliana* lines with modified expression (HAC1-OE and HAC1-RNAi)and control (WT).

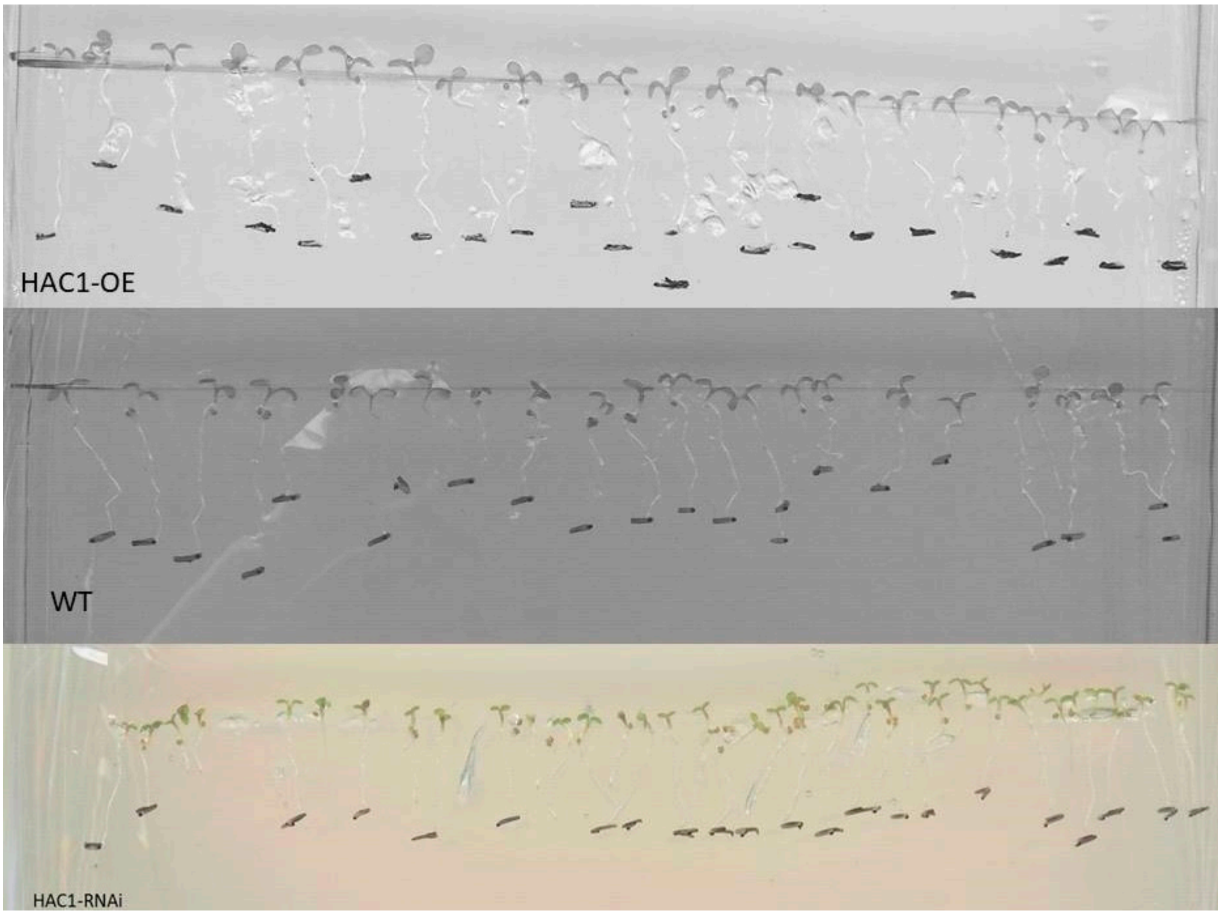

Figure. S2. Age of rosettes from *A. thaliana* transgenic and control plants for collection of samples from leaf area measurement.

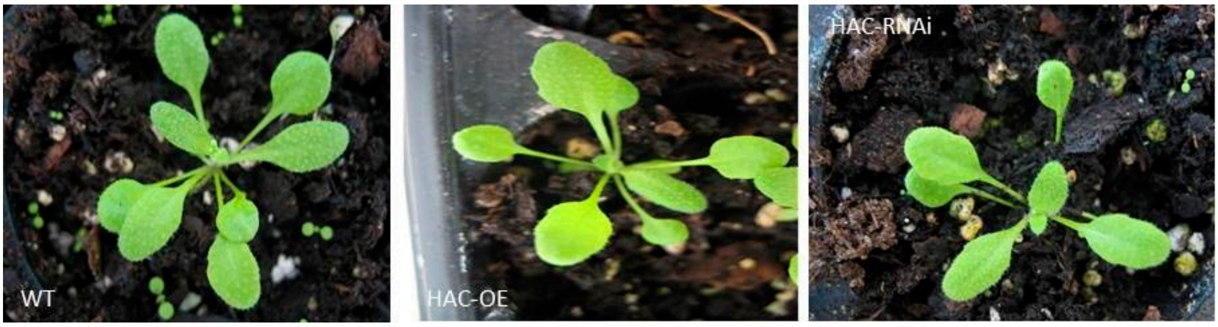

Figure S3. Samples for leaf area measurement.

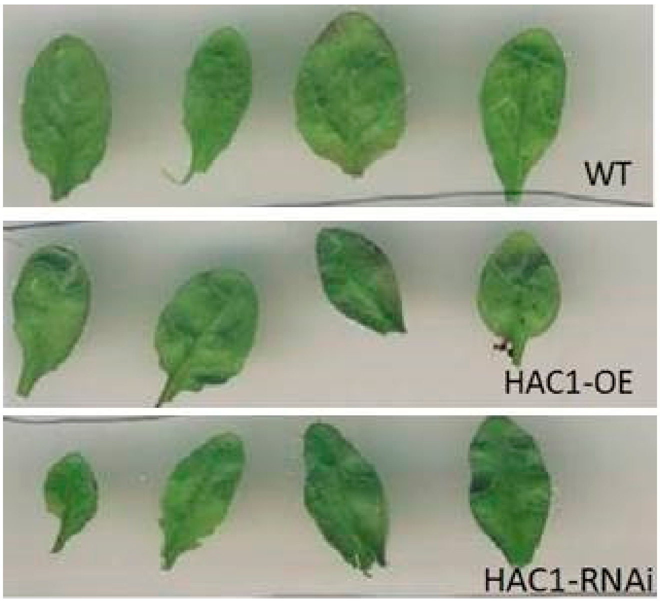

Figure S4. *A. thaliana* plants (WT, HAC1-OE and HAC-RNAI) before low temperature (4°C) stress.

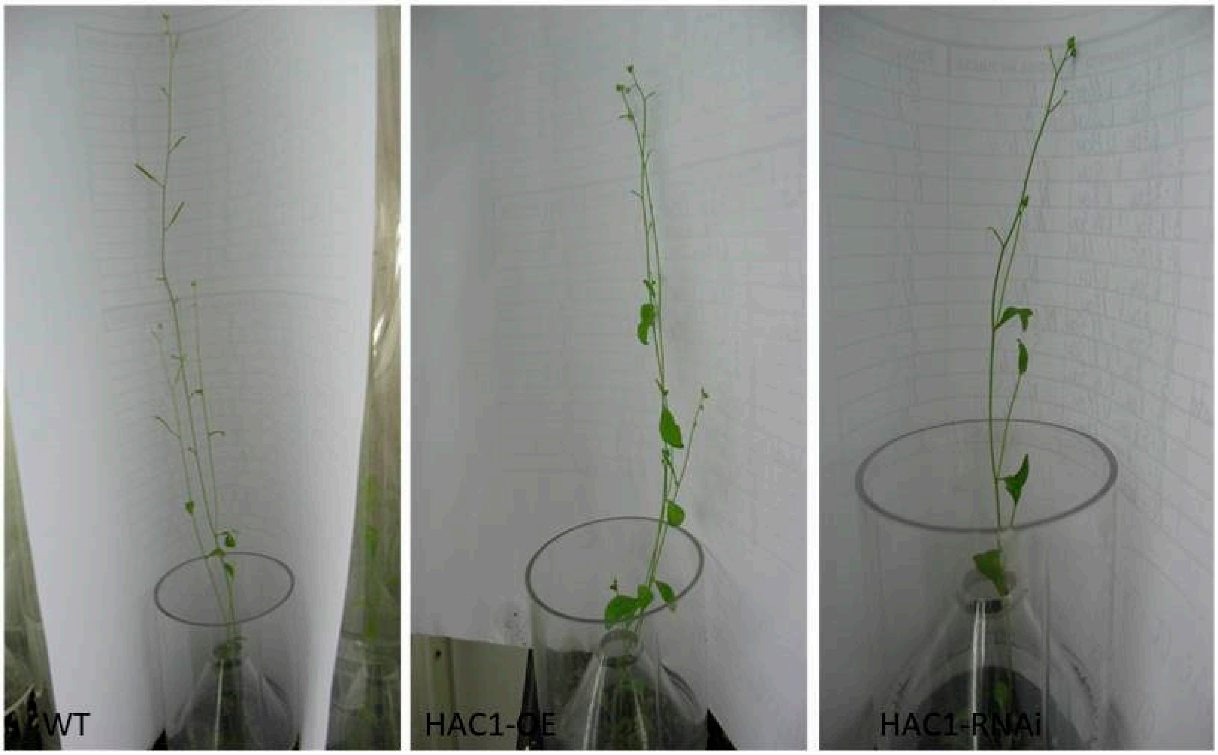

Figure S5. Amplification products. A- amplicon of HAC1-OE sample; B- amplicon of HAC1-RNAi sample.

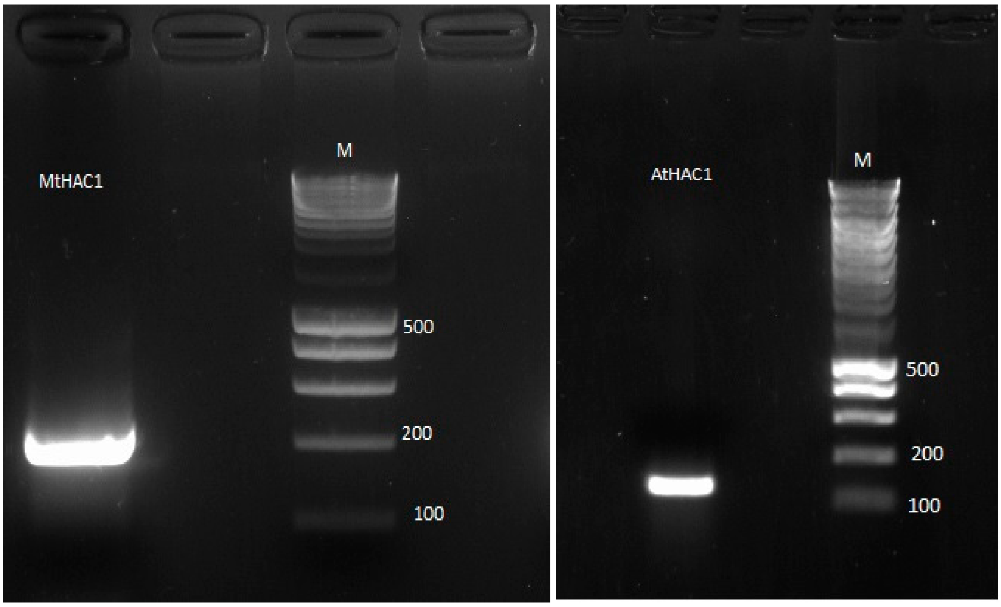

Table S1. Primers used in qRT-PCR analyses.

MtHAC1- forward	5' TTCCTCCGACAAGCAGCTAT 3'
MtHAC1- reverse	5' CTATGCGATGACACCCACTG 3'
AtHAC1- forward	5’GGTGATGCTACAATGGCTGG 3’
AtHAC1- reverse	5’ATGTTCCCTCCAGACCCAAG 3’
At actin - forward	5' TGCCAATCTACGAGGGTTTC 3'
At actin - reverse	5' TTCTCGATGGAAGAGCTGGT 3'
At ubiquitin- forward	5' GTCGACCCTTCACTTGGTGT 3'
At ubiquitin- reverse	5' CCTTGACGTTGTCAATGGTG 3'

Table S2. Content and quantity of detected polar and non-polar free metabolites in µg/g DW in non-treated and treated samples.

Name	WT 0h	WT 72h NaCl	HAC1-RNAi 0h	HAC1 RNAi-72h NaCl	HAC1-OE 0h	HAC1-OE 72h NaCl

Amino acids						
Alanine	7.33 ± 0.13	8.06 ± 0.21	9.05 ± 0.22	9.95 ± 0.14	6.52 ± 0.11	6.95 ± 0.10
**Valine**	64.08 ± 2.21	70.49 ± 1.12	79.11 ± 3.10	87.02 ± 2.50	57.03 ± 1.12	60.74 ± 1.42
**Leucine**	59.28 ± 1.58	65.21 ± 2.10	73.19 ± 2.50	80.50 ± 1.90	52.76 ± 1.84	56.19 ± 1.97
**Isoleucine**	43.19 ± 0.56	47.51 ± 0.84	53.32 ± 0.69	58.65 ± 0.57	38.44 ± 0.78	40.94 ± 0.35
Proline	22.05 ± 0.24	24.26 ± 0.12	27.22 ± 0.32	29.94 ± 0.65	19.64 ± 0.14	20.90 ± 0.23
Glycine	7.84 ± 0.10	8.63 ± 0.09	9.68 ± 0.07	10.65 ± 0.11	6.98 ± 0.13	7.43 ± 0.12
**Serine**	106.42 ± 3.54	117.06 ± 4.10	131.38 ± 3.87	144.51 ± 2.98	94.71 ± 3.15	100.87 ± 3.99
**Threonine**	55.31 ± 1.98	60.84 ± 1.54	68.28 ± 1.62	75.11 ± 1.71	49.23 ± 1.56	52.43 ± 1.39
Aspartic acid	35.20 ± 1.25	38.72 ± 1.35	43.46 ± 1.11	47.80 ± 1.41	31.33 ± 1.51	33.37 ± 1.28
Methionine	10.55 ± 0.51	11.61 ± 0.47	13.03 ± 0.35	14.33 ± 0.63	9.39 ± 0.45	10.00 ± 0.48
**Pyroglutamic acid**	96.49 ± 4.11	106.14 ± 2.98	119.12 ± 3.15	131.03 ± 4.25	85.88 ± 3.87	91.46 ± 3.12
Cisteine	8.85 ± 0.14	9.74 ± 0.12	10.93 ± 0.34	12.02 ± 0.58	7.88 ± 0.45	8.39 ± 0.65
**Asparagine**	50.27 ± 1.98	55.30 ± 1.85	62.06 ± 1.56	68.26 ± 1.87	44.74 ± 1.99	47.65 ± 1.69
Glutamic acid	23.72 ± 1.51	26.09 ± 0.87	29.28 ± 0.98	32.21 ± 0.67	21.11 ± 0.66	22.48 ± 0.97
Phenylalanine	11.09 ± 0.32	12.20 ± 0.14	13.69 ± 0.21	15.06 ± 0.31	9.87 ± 0.25	10.51 ± 0.13
**Glutamine**	108.45 ± 3.54	119.30 ± 4.10	133.88 ± 3.87	147.27 ± 2.98	96.52 ± 3.15	102.80 ± 3.99
**Arginine**	60.88 ± 2.21	66.97 ± 1.12	75.16 ± 3.10	82.67 ± 2.50	54.18 ± 1.12	57.71 ± 1.42
Lysine	20.17 ± 0.24	22.19 ± 0.12	24.90 ± 0.32	27.39 ± 0.65	17.95 ± 0.14	19.12 ± 0.23
Tyrosine	12.28 ± 0.51	13.51 ± 0.47	15.16 ± 0.35	16.68 ± 0.63	10.93 ± 0.45	11.64 ± 0.48
Histidine	6.90 ± 0.13	7.59 ± 0.21	8.52 ± 0.12	9.37 ± 0.14	6.14 ± 0.11	6.54 ± 0.10
Tryptophane	11.46 ± 0.52	12.60 ± 0.56	14.14 ± 0.35	15.56 ± 0.64	10.19 ± 0.45	10.86 ± 0.48

Organic acids

Pyruvic acid	2.21 ± 0.14	2.43 ± 0.11	2.73 ± 0.24	3.00 ± 0.58	1.97	2.10
Malonic acid	4.67 ± 0.98	5.13 ± 0.24	5.76 ± 0.66	6.34 ± 0.12	4.15 ± 0.46	4.42 ± 0.54

Succinic acid	16.03 ± 1.13	17.63 ± 1.10	19.79 ± 1.34	21.77 ± 1.14	14.27 ± 1.78	15.19 ± 1.66
Glyceric acid	5.37 ± 0.57	5.91 ± 0.68	6.63 ± 0.84	7.29 ± 0.87	4.78 ± 0.64	5.09 ± 0.46
Fumaric acid	14.05 ± 1.22	15.46 ± 1.45	17.35 ± 1.68	19.08 ± 1.25	12.51 ± 1.46	13.32 ± 1.32
Malic acid	19.63 ± 2.10	21.59 ± 1.88	24.23 ± 1.98	26.66 ± 1.34	17.47 ± 1.84	18.61 ± 1.55
GABA	17.18 ± 1.54	18.90 ± 1.64	21.21 ± 1.42	23.33 ± 1.64	15.29 ± 1.22	16.29 ± 1.84
**Gluconic acid**	75.32 ± 3.64	82.85 ± 1.45	92.98 ± 2.12	102.28 ± 2.58	67.04 ± 2.98	71.39 ± 1.28

Mono- and dicarbohydrates, sugar alcohols

Xylose	105.13 ± 5.86	115.65 ± 3.47	129.79 ± 3.45	142.77 ± 4.06	93.57 ± 2.42	99.65 ± 2.22
Arabinose	90.96 ± 3.46	100.06 ± 2.98	112.29 ± 2.68	123.52 ± 2.65	80.96 ± 2.98	86.22 ± 3.64
**Fructose isomer**	431.41 ± 22.31	474.55 ± 20.88	532.58 ± 24.45	585.83 ± 20.66	383.95 ± 22.66	408.91 ± 24.57
**Fructose isomer**	330.04 ± 21.98	363.04 ± 19.66	407.43 ± 18.36	448.18 ± 16.47	293.74 ± 18.48	312.83 ± 19.88
Galactose isomer	109.02 ± 3.44	119.92 ± 3.56	134.59 ± 4.12	148.05 ± 4.02	97.03 ± 4.86	103.34 ± 5.14
**Glucose isomer**	495.39 ± 32.11	544.93 ± 28.45	611.56 ± 34.18	672.71 ± 31.88	440.90 ± 32.42	469.56 ± 30.88
Galactose isomer	70.15 ± 2.98	77.17 ± 1.98	86.60 ± 2.24	95.26 ± 2.54	62.43 ± 3.15	66.49 ± 4.02
**Glucose isomer**	191.06 ± 4.12	210.17 ± 4.15	235.87 ± 5.18	259.46 ± 6.38	170.05 ± 5.88	181.10 ± 6.14
myo-Inositol	96.24 ± 3.88	105.87 ± 2.66	118.81 ± 3.64	130.69 ± 4.12	85.65 ± 3.14	91.22 ± 3.58
**Sucrose isomer**	813.46 ± 41.15	894.81 ± 40.64	1004.22 ± 45.28	1104.64 ± 48.24	723.98 ± 35.88	771.04 ± 34.18
Sucrose isomer	18.90 ± 1.25	20.79 ± 1.87	23.33 ± 1.54	25.67 ± 1.57	16.82 ± 1.54	17.91 ± 1.64

Fatty acids

**Palmitic acid**	380.37 ± 22.31	418.41 ± 24.11	469.57 ± 35.12	516.53 ± 28.14	338.53 ± 32.12	360.54 ± 28.45
**Linoleic acid**	114.71 ± 3.54	126.18 ± 4.10	141.61 ± 3.87	155.77 ± 2.98	102.09 ± 3.15	108.73 ± 3.99
**Oleic acid**	68.04 ± 2.21	74.85 ± 1.12	84.00 ± 3.10	92.40 ± 2.50	60.56 ± 1.12	64.49 ± 1.42
**Linolenic acid**	491.18 ± 32.11	540.30 ± 25.95	606.36 ± 30.25	667.00 ± 28.15	437.15 ± 26.33	465.56 ± 27.45
**Stearic acid**	354.26 ± 23.12	389.69 ± 20.99	437.34 ± 32.12	481.07 ± 27.66	315.29 ± 22.45	335.79 ± 26.13
